# Supplementary material for: Metabolic biomarker profiling for identification of susceptibility to severe pneumonia and COVID-19 in the general population
Source: eLife. 2021 May 4;10:e63033. doi: 10.7554/eLife.63033 (PMC8172246; doi:10.7554/eLife.63033)
Supplement: Supplementary file 1. — The table indicates the weights for the 25 biomarkers that were selected in derivation of the multi-biomarker infectious disease score, based on optimising prediction for severe pneumonia using logistic regression with LASSO in the derivation half of the study population (n = 52,573). Each biomarker was scaled to SD-units prior to the analyses. The infectious disease score was then calculated as β1×X1 + β2×X2 + … + β25×X25, with Xi denoting the SD-standardised biomarker level for the ith biomarker and βi denoting the coefficient from the multi-biomarker logistic regression model. DHA indicates docosahexaenoic acid; MUFA: monounsaturated fatty acids; PUFA: polyunsaturated fatty acids; SFA: saturated fatty acids. [file elife-63033-supp1.docx]

**Supplementary File 1. Weights of the biomarkers included in the multi-biomarker infectious disease score derived using LASSO regression.**

| **Biomarker (SD units)** | **β-weight in the**  **‘Infectious disease score’** |
| --- | --- |
| Glycoprotein acetyls (GlycA) | 0.3713 |
| DHA % | 0.2533 |
| Creatinine | 0.2170 |
| MUFA | 0.1693 |
| Apolipoprotein B / Apolipoprotein A1 | 0.1388 |
| Tyrosine | 0.1375 |
| Isoleucine | 0.1091 |
| SFA % | 0.0965 |
| Glucose | 0.0928 |
| Lactate | 0.0772 |
| Omega-6/Omega-3 | 0.0642 |
| Phenylalanine | 0.0289 |
| Total cholesterol | -0.0116 |
| Omega-6 % | -0.0493 |
| Alanine | -0.0498 |
| PUFA | -0.0578 |
| Glycine | -0.0648 |
| Histidine | -0.0987 |
| PUFA % | -0.1404 |
| Valine | -0.1812 |
| Leucine | -0.1844 |
| Albumin | -0.1914 |
| Omega-3 | -0.2208 |
| LDL cholesterol | -0.2466 |
| Triglycerides | -0.2652 |

The table indicates the weights for the 25 biomarkers that were selected in derivation of the multi-biomarker infectious disease score, based on optimising prediction for severe pneumonia using logistic regression with LASSO in the derivation half of the study population (n=52573). Each biomarker was scaled to SD-units prior to the analyses. The infectious disease score was then calculated as β1*X*1 + β2*X*2 + … + β25*X*25, with *Xi* denoting the SD-standardized biomarker level for the *i*th biomarker and β*i* denoting the coefficient from the multi-biomarker logistic regression model. DHA indicates docosahexaenoic acid; MUFA: monounsaturated fatty acids; PUFA: polyunsaturated fatty acids; SFA: saturated fatty acids.
